# Tumor of the Breast—Diagnostic Value of the Discharge from the Nipple

**Published:** 1854-03

**Authors:** 


Tumor of the Breast—Diagnostic value of the Discharge from the Nipple.—Dr. Richard reports, in the Revue Medico-Chirurgicale, six cases of Tumors of the Breast, in which he observed a discharge from the nipple. This discharge varied very much in character, from the appearance of pure blood to that of clear water. It was very abundant in certain cases, flowing out spontaneously; whilst in others there was only an oozing, or a drop or two would escape, on pressure being made. In spite of this diversity of aspect, the liquid could be considered as an habitual lactiform secretion, mixed or not, with blood. Another important result was, that all the tumors had the same nature, which was partial hypertrophy of the mammary gland, but the form and degree of the malady varied. In cases of long standing, where there was softening of the gland, the blood was found in a kind of cyst, in those more recent, the lacteous secretion predominated. This discharge appears to be peculiar to simple hypertrophy of the mammary gland; for, in twenty-seven cases of cancer of the breast, confirmed by examination with the microscope, not one showed a discharge from the nipple. The author concludes, from his observations, that the discharge from the nipple is a frequent symptom of partial hypertrophy of the mammary gland, and that its existence is a favorable prognostic in tumors of the breast.—*Charleston Journal.*

